# Supplementary material for: A Multiassessment and Multiprofessional Agents Approach for Medical Chatbot Risk Estimation: Development and Evaluation Study
Source: JMIR Med Inform. 2026 May 15;14:e80416. doi: 10.2196/80416 (PMC13221620; doi:10.2196/80416)
Supplement: Multimedia Appendix 3 [file medinform_v14i1e80416_app3.docx]

## Multimedia Appendix 3: External Documents

Non-Japanese source

Wikipedia (search phrases):

- Values, Etiquette, Health care system, Health, Health Law

US and EU health-related external evidence

- Over-the-Counter (OTC) Medical Devices: Considerations for Device Manufacturers [30]
- Guidance for Over-the-Counter (OTC) Human Chorionic Gonadotropin (hCG) 510(k)s - Guidance for Industry and FDA Reviewers/Staff [31]
- Latex Condoms for Men - Information for 510(k) Premarket Notifications: Use of Consensus Standards for Abbreviated Submissions [32]
- Menstrual Tampons and Pads: Information for Premarket Notification Submissions (510(k)s) - Guidance for Industry and FDA Staff [33]
- Labeling Requirements - Over-The-Counter (Non-Prescription) Medical Devices [34]
- European Agency for Safety and Health at Work (EU-OSHA), and repealing Council Regulation (EC) No 2062/94 [35]
- Decision (eu) 2021/75 of the european parliament and of the council of 25 November 2020 on the mobilisation of the European Union Solidarity Fund to provide assistance to Croatia and Poland in relation to a natural disaster and to provide for the payment of advances to Croatia, Germany, Greece, Hungary, Ireland, Portugal and Spain in relation to a public health emergency [36]
- Directive 2004/23/EC of the European Parliament and of the Council of 31 March 2004 on setting standards of quality and safety for the donation, procurement, testing, processing, preservation, storage and distribution of human tissues and cells [37]
- Regulation (EU) 2017/745 of the European Parliament and of the Council of 5 April 2017 on medical devices, amending Directive 2001/83/EC, Regulation (EC) No 178/2002 and Regulation (EC) No 1223/2009 and repealing Council Directives 90/385/EEC and 93/42/EEC (Text with EEA relevance.) [38]
- Directive 2011/24/eu of the european parliament and of the council of 9 March 2011 on the application of patients’ rights in cross-border healthcare [39]
- Regulation (EU) 2021/2282 of the European Parliament and of the Council of 15 December 2021 on health technology assessment and amending Directive 2011/24/EU (Text with EEA relevance) [40]
- Directive 2000/54/EC of the European Parliament and of the Council of 18 September 2000 on the protection of workers from risks related to exposure to biological agents at work (seventh individual directive within the meaning of Article 16(1) of Directive 89/391/EEC) [41]
- Regulation (EU) 2016/429 of the European Parliament and of the Council of 9 March 2016 on transmissible animal diseases and amending and repealing certain acts in the area of animal health (‘Animal Health Law’) (Text with EEA relevance) [42]

Japanese source

Wikipedia (search phrases):

- Japanese values, Etiquette in Japan, Health care system in Japan, Health in Japan, Law of Japan

Japanese Law Translation:

- Health Insurance Act (Act No. 70 of 1922) [43]
- Community Health Act (Act No. 101 of 1947) [44]
- Medical Practitioners' Act (Act No. 201 of 1948) [45]
- Act on Public Health Nurses, Midwives, and Nurses (Act No. 203 of 1948) [46]
- Medical Care Act (Act No. 205 of 1948) [47]
- Regulations for Enforcement of the Medical Practitioners Act (Order of the Ministry of Health and Welfare No. 47 of 1948) [48]
- Order for Enforcement of the Act on Securing Quality, Efficacy and Safety of Products Including Pharmaceuticals and Medical Devices (Cabinet Order No. 11 of 1961) [49]
- Act on Securing Quality, Efficacy and Safety of Products Including Pharmaceuticals and Medical Devices (Act No. 145 of 1960) [50]
- Industrial Safety and Health Act (Act No. 57 of 1972) [51]
- Ordinance on Industrial Safety and Health (Order of the Ministry of Labour No. 32 of 1972) [52]
- Long-Term Care Insurance Act (Act No. 123 of 1997) [53]
- Act on the Prevention of Infectious Diseases and Medical Care for Patients with Infectious Diseases (Act No. 114 of 1998) [54]

Ministry of Health, Labour and Welfare:

- Heatstroke Prevention Manual [55]
- Enforcement of Amended Organ Transplantation Law [56]
- Verification Committee Concerning Hansen’s Disease Problem [57]
- Basic guidelines for promotion of control measures for hepatitis [58]
- Implementation Manual for the National Epidemiological Surveillance of Infectious Diseases Program [59]
- Outline of the Act on the Partial Revision of the Health Promotion Act (No. 78 of 2018) [60]
- The Act on Assistance Dogs for Physically Disabled Persons [61]
- Assistance Dog Information BOOK Ministry of Health, Labour and Welfare [62]
- Long-Term Care Insurance System of Japan [63]
- Outline of the revision of the Long-Term Care Insurance System, etc. to strengthen the Community-based Integrated Care System in 2017-18 [64]
- Establishing the Community-based Integrated Care System [65]
