# Supplementary material for: A Multiassessment and Multiprofessional Agents Approach for Medical Chatbot Risk Estimation: Development and Evaluation Study
Source: JMIR Med Inform. 2026 May 15;14:e80416. doi: 10.2196/80416 (PMC13221620; doi:10.2196/80416)
Supplement: Multimedia Appendix 4 [file medinform_v14i1e80416_app4.docx]

## Multimedia Appendix 4: Retrieval-Augmented Generation (RAG) using non-Japanese and Japanese external evidence.

Table S1. RAG preliminary exploration using non-Japanese external evidence.

| **System** | **Macro *F*_1_-score** **and Joint Accuracy Performance** | | | |
| --- | --- | --- | --- | --- |
|  | **Medical Risk** | **Ethical Risk** | **Legal Risk** | **Joint Accuracy (%)** |
| RAG only using non-Japanese external evidence ^a^ | 0.400 | 0.476 | 0.471 | 19.8 (25/126) |
| RAG (MA + MPA) using non-Japanese external evidence ^b^ | 0.623 | 0.867 | 0.878 | 57.1 (72/126) |

^a^ using RAG without multiassessment (MA) and multiprofessional agents (MPA)

^b^ utilizing the proposed framework (multiassessment (MA) and multiprofessional agents (MPA)), showing final assessment (MA3) results
