# Supplementary material for: A Multiassessment and Multiprofessional Agents Approach for Medical Chatbot Risk Estimation: Development and Evaluation Study
Source: JMIR Med Inform. 2026 May 15;14:e80416. doi: 10.2196/80416 (PMC13221620; doi:10.2196/80416)
Supplement: Multimedia Appendix 5 [file medinform_v14i1e80416_app5.docx]

## Multimedia Appendix 5: CIs and paired macro *F*_1_-score difference (Δ).

Table S1: This table reports the average macro *F*_1_-score and joint accuracy in percentage (%) with 95% CI estimate across systems.

| **Systems** | **Macro *F*_1_-score and Joint Accuracy Performance across systems and assessment stage** | | | | | |
| --- | --- | --- | --- | --- | --- | --- |
|  | **Average Macro *F*_1_-score (95% CI)^b^** | | | **Joint Accuracy % (95% CI)^b^** | | |
|  | **MA1** | **MA2** | **MA3** | **MA1** | **MA2** | **MA3** |
| BERT^a^ | - | - | 0.480 (0.427 - 0.532) | - | - | 50.0 (41.3–57.9) |
| BioClinicalBERT^a^ | - | - | 0.521 (0.470 - 0.570) | - | - | 37.3 (28.6–45.2) |
| Baseline | 0.538 (0.479 - 0.595) | 0.752 (0.676 - 0.812) | 0.739 (0.662 - 0.795) | 41.3 (32.5–50.0) | 56.3 (47.6–64.3) | 54.0 (45.2–62.7) |
| Enhanced Prompt | 0.530 (0.484 - 0.573) | 0.706 (0.622 - 0.769) | 0.743 (0.651 - 0.809) | 38.1 (29.4–46.8) | 50.0 (42.1–58.7) | 54.8 (46.0–62.7) |
| Embedding-based search | 0.514 (0.471 - 0.561) | 0.725 (0.654 - 0.785) | 0.790 (0.720 - 0.842) | 40.5 (31.7–49.2) | 51.6 (42.9–60.3) | 57.9 (49.2–65.9) |
| RAG (Full system, non-ablated) | 0.573 (0.521 - 0.623) | 0.767 (0.702 - 0.823) | 0.800 (0.733 - 0.852) | 42.9 (34.1–50.8) | 55.6 (46.8–63.5) | 60.3 (51.6–68.3) |

^a^BERT systems are rerun using the same 100 train/126 test split. These models do not necessarily use the multiassessment (MA) and multiprofessional agents (MPA) framework.

^b^MA: multiassessment

Table S2: This table reports the paired macro *F*_1_-score and joint accuracy in percentage (%) of systems using a multiassessment (MA) approach. It provides a 95% CI estimate for the change in macro *F*_1_-score between the multiassessment phases (MA1 vs. MA2 vs. MA3).

| **Systems** | **Paired macro *F*_1_-score difference (Δ) and joint accuracy of MA1 vs MA2 vs MA3** | | | | | |
| --- | --- | --- | --- | --- | --- | --- |
|  | **Δ Macro *F*_1_-score (95% CI)^ab^** | | | **Δ Joint Accuracy % (95% CI)^ab^** | | |
|  | **MA1 vs MA2** | **MA2 vs MA3** | **MA1 vs MA3** | **MA1 vs MA2** | **MA2 vs MA3** | **MA1 vs MA3** |
| Baseline | 0.214 (0.138 to 0.290) | -0.013 (-0.059 to 0.029) | 0.201 (0.133 to 0.260) | 15.1 (3.2 to 26.2) | -2.4 (-5.6 to 0.8) | 12.7 (1.6 to 23.8) |
| Enhanced Prompt | 0.176 (0.092 to 0.257) | 0.037 (0.014 to 0.064) | 0.213 (0.122 to 0.292) | 11.9 (0.8 to 23.8) | 4.8 (1.6 to 8.7) | 16.7 (4.8 to 28.6) |
| Embedding -based search | 0.211 (0.144 to 0.273) | 0.065 (0.031 to 0.102) | 0.276 (0.212 to 0.334) | 11.1 (-0.8 to 23.0) | 6.3 (2.4 to 11.1) | 17.5 (5.6 to 29.4) |
| RAG (Full system, non-ablated) | 0.194 (0.129 to 0.253) | 0.033 (0.010 to 0.065) | 0.226 (0.157 to 0.289) | 12.7 (0.8 to 25.4) | 4.8 (1.6 to 8.7) | 17.5 (5.5 to 30.2) |

^a^Differences (Δ) were computed as Δ = MA2 - MA1, Δ = MA3 - MA2 and Δ = MA3 - MA1

^b^MA: multiassessment

Table S3. This table reports paired changes in macro *F*_1_-score and joint accuracy in percentage (%) for separate comparisons of RAG vs baseline and RAG vs enhanced prompt system.

| **Systems** | **Paired macro *F*_1_-score difference (Δ) and joint accuracy of RAG vs Non-RAG** | |
| --- | --- | --- |
|  | **Δ Macro *F*_1_-score (95% CI)^a^** | **Δ Joint Accuracy % (95% CI)^a^** |
| Baseline vs RAG ^a^ | 0.037 (0.003 to 0.074) | 2.4 (-2.9 to 7.7) |
| Enhanced prompt vs RAG ^a^ | 0.054 (0.010 to 0.100) | 5.3 (0.3 to 10.6) |

^a^Paired performance differences (Δ) estimated using the nonparametric bootstrap and computed as Δ = RAG - non-RAG. Comparisons are reported separately for baseline vs RAG and enhanced prompt vs RAG.
