# Supplementary material for: A Multiassessment and Multiprofessional Agents Approach for Medical Chatbot Risk Estimation: Development and Evaluation Study
Source: JMIR Med Inform. 2026 May 15;14:e80416. doi: 10.2196/80416 (PMC13221620; doi:10.2196/80416)
Supplement: Multimedia Appendix 6 [file medinform_v14i1e80416_app6.docx]

## Multimedia Appendix 6: Accuracy, precision, and recall results.

Table S1. Accuracy, precision and recall reports across systems, risk domains and assessment phase.

| **Systems** | **MA Phase^a^** | **Accuracy^b^** | | | **Precision^bc^** | | | **Recall^bc^** | | |
| --- | --- | --- | --- | --- | --- | --- | --- | --- | --- | --- |
|  |  | **M** | **E** | **L** | **M** | **E** | **L** | **M** | **E** | **L** |
| Baseline | MA1 | 0.635 | 0.746 | 0.698 | 0.615 | 0.557 | 0.483 | 0.596 | 0.690 | 0.477 |
|  | MA2 | 0.643 | 0.960 | 0.881 | 0.689 | 0.825 | 0.757 | 0.678 | 0.862 | 0.792 |
|  | MA3 | 0.627 | 0.944 | 0.889 | 0.687 | 0.764 | 0.771 | 0.668 | 0.854 | 0.819 |
| Enhanced prompt | MA1 | 0.611 | 0.746 | 0.698 | 0.587 | 0.524 | 0.516 | 0.576 | 0.573 | 0.523 |
|  | MA2 | 0.627 | 0.937 | 0.881 | 0.696 | 0.733 | 0.757 | 0.671 | 0.733 | 0.769 |
|  | MA3 | 0.651 | 0.952 | 0.913 | 0.710 | 0.817 | 0.835 | 0.691 | 0.742 | 0.787 |
| Embedding-based search | MA1 | 0.611 | 0.746 | 0.722 | 0.586 | 0.524 | 0.471 | 0.573 | 0.573 | 0.468 |
|  | MA2 | 0.627 | 0.913 | 0.913 | 0.706 | 0.691 | 0.825 | 0.674 | 0.837 | 0.810 |
|  | MA3 | 0.651 | 0.968 | 0.937 | 0.719 | 0.867 | 0.902 | 0.694 | 0.867 | 0.824 |
| RAG | MA1 | 0.651 | 0.746 | 0.778 | 0.635 | 0.541 | 0.579 | 0.613 | 0.631 | 0.593 |
|  | MA2 | 0.651 | 0.960 | 0.905 | 0.719 | 0.814 | 0.812 | 0.694 | 0.921 | 0.782 |
|  | MA3 | 0.675 | 0.976 | 0.921 | 0.732 | 0.885 | 0.862 | 0.714 | 0.929 | 0.792 |

^a^MA: multiassessment; MPA: multiprofessional agents; EK: external knowledge.

^b^M: medical risk; E: ethical risk; L: legal risk

^c^Reports macro precision and recall where precision_macro_ = precision_false_ + precision_true_ / 2 and recall_macro_ = recall_false_ + recall_true_ / 2
