# Supplementary material for: A Multiassessment and Multiprofessional Agents Approach for Medical Chatbot Risk Estimation: Development and Evaluation Study
Source: JMIR Med Inform. 2026 May 15;14:e80416. doi: 10.2196/80416 (PMC13221620; doi:10.2196/80416)
Supplement: Multimedia Appendix 9 [file medinform_v14i1e80416_app9.docx]

## Multimedia Appendix 9: Confusion Matrices


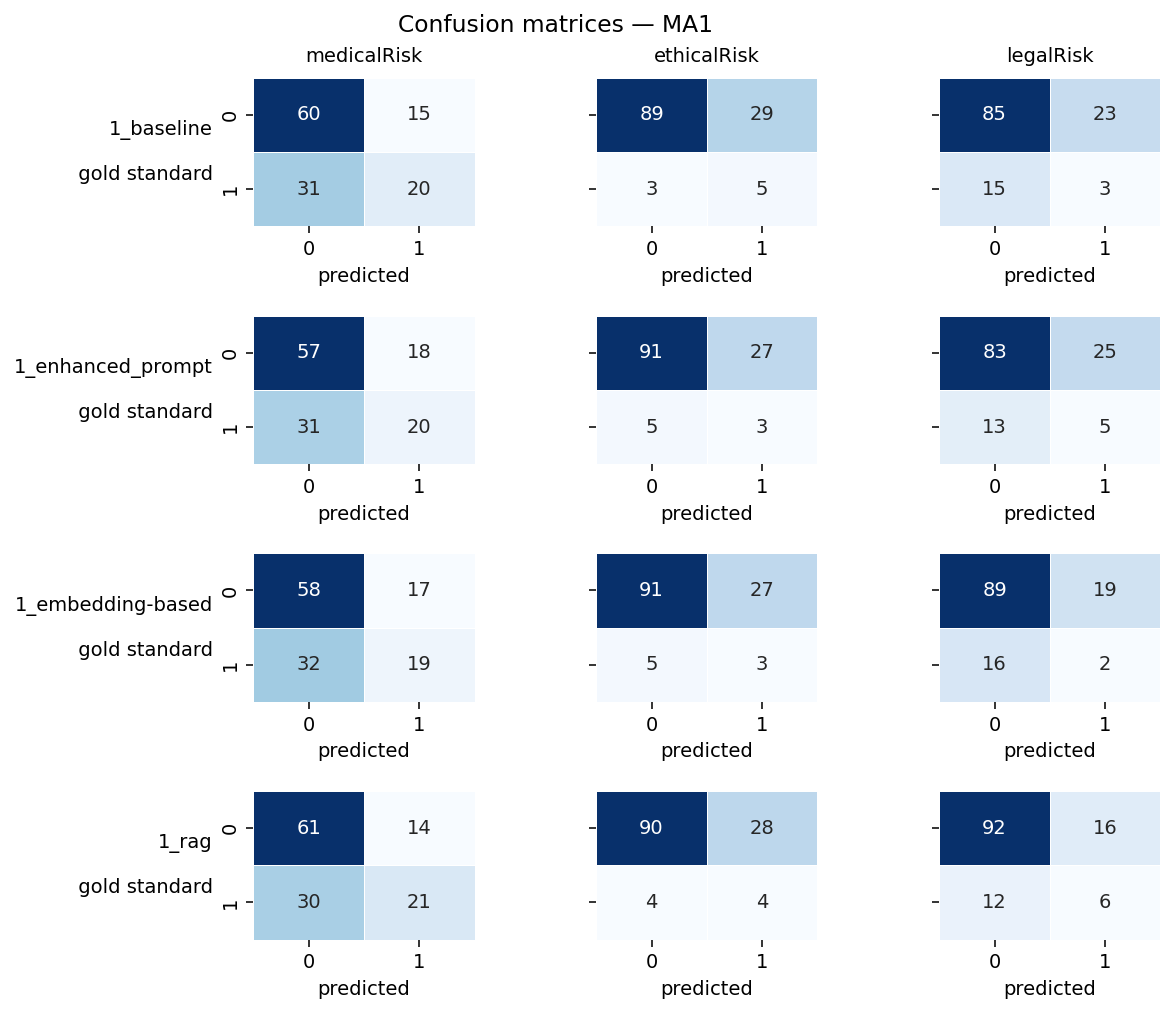

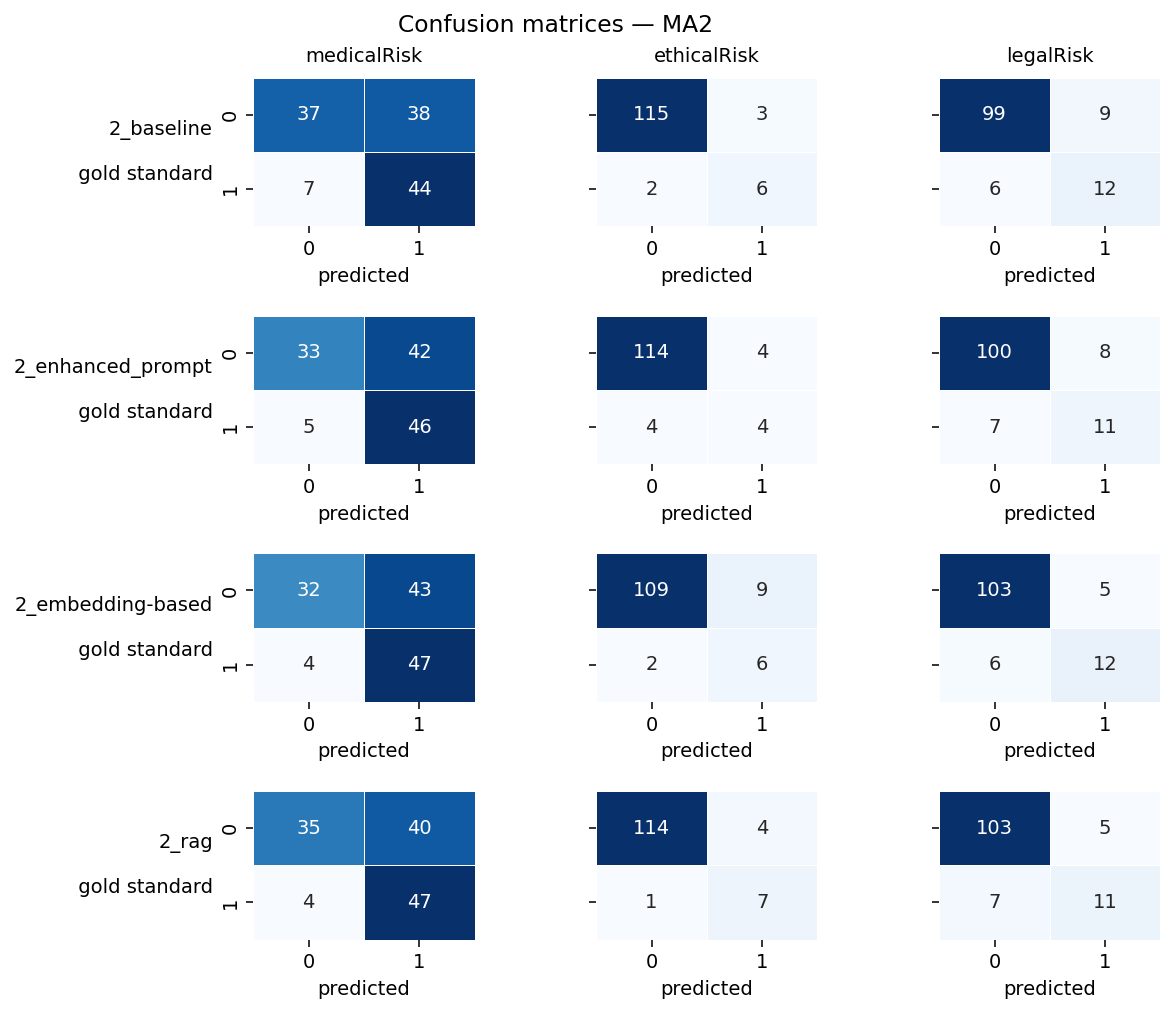


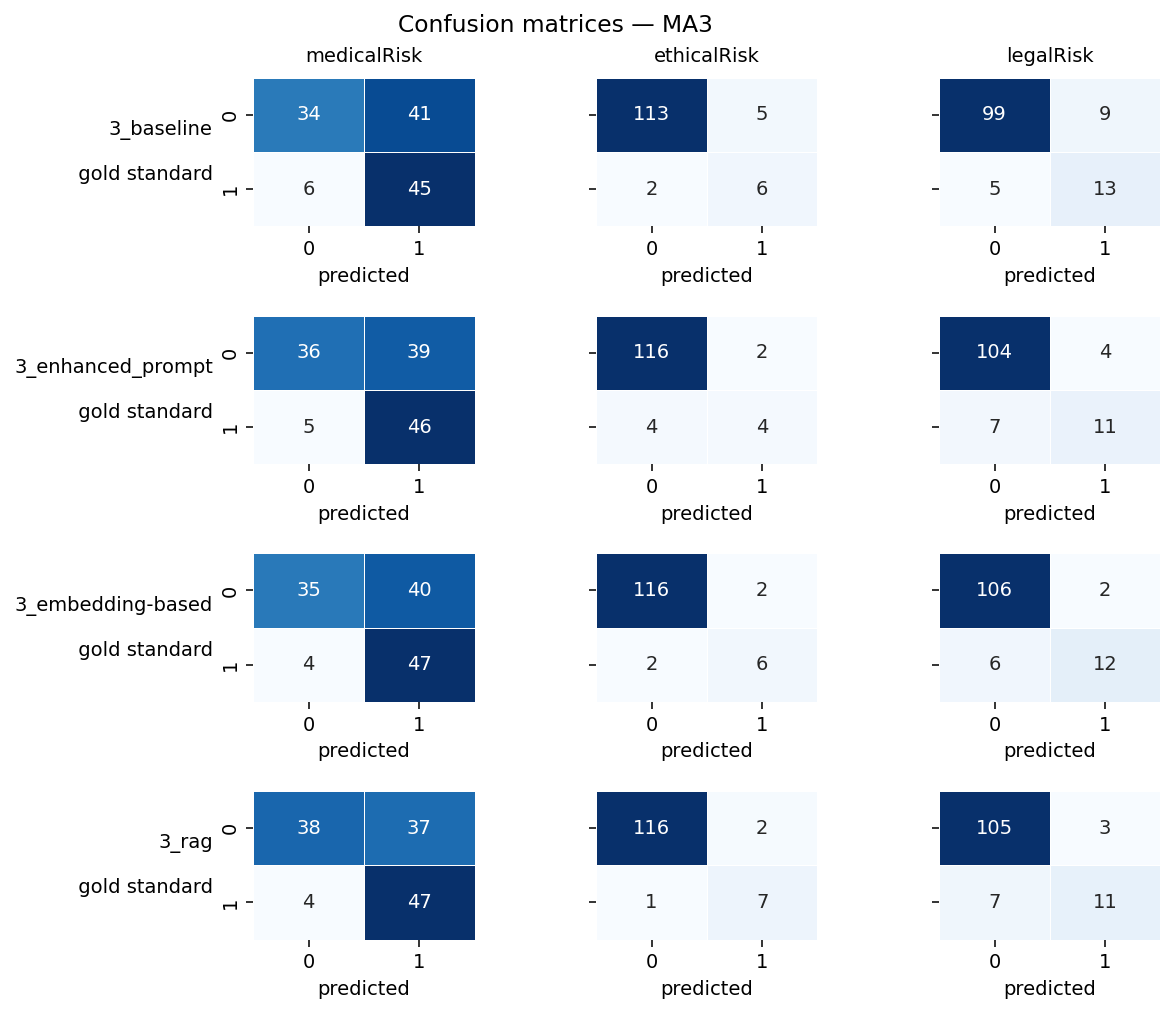


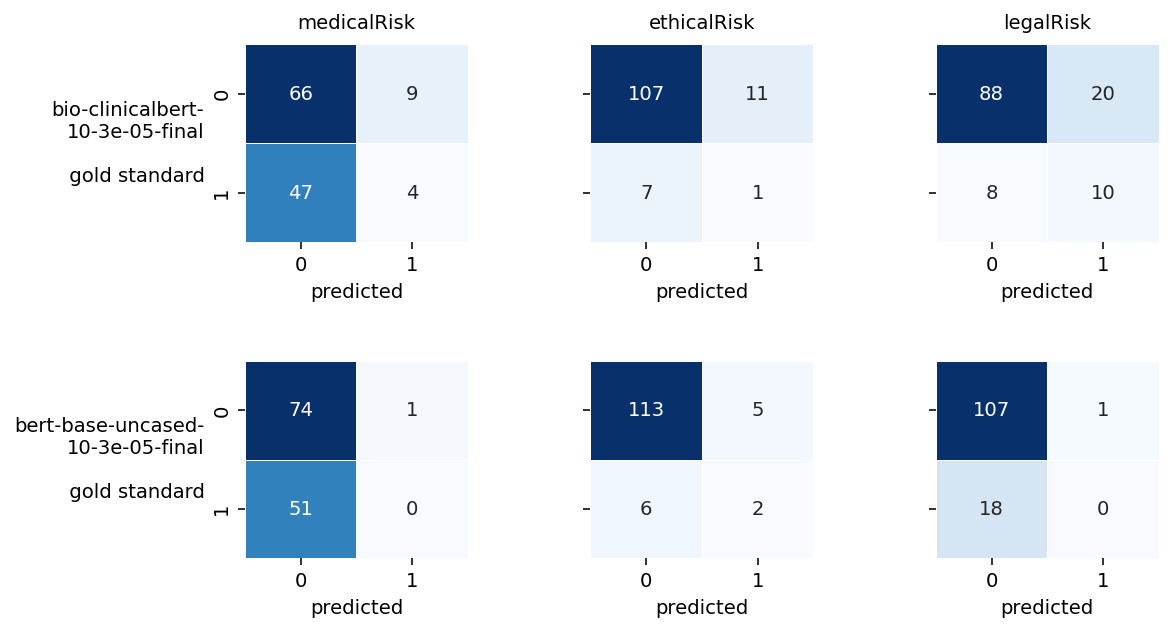


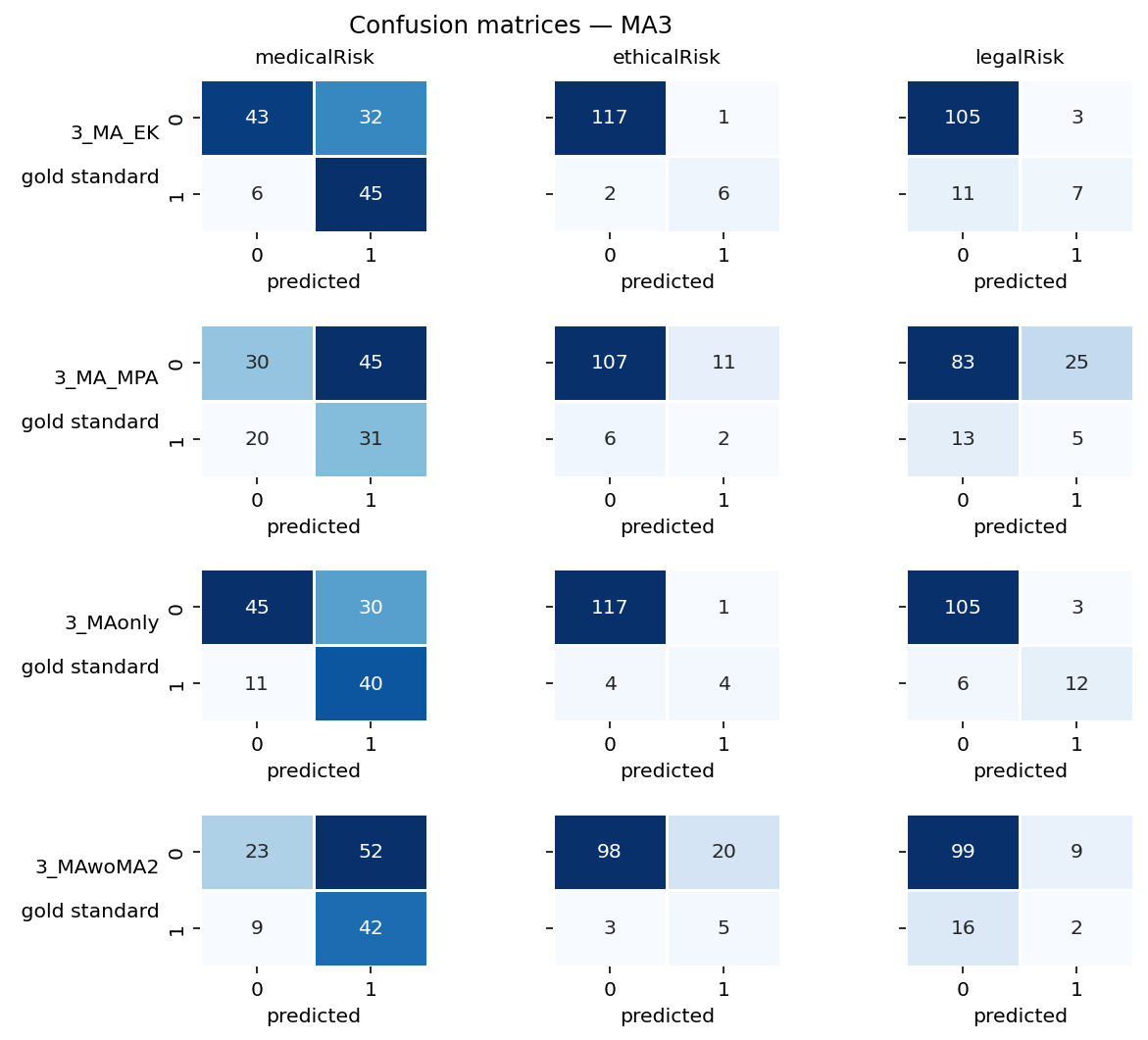


Multimedia Appendix Figure 1. Confusion matrix across systems, multiassessment phase and domains
