# Supplementary material for: A Multiassessment and Multiprofessional Agents Approach for Medical Chatbot Risk Estimation: Development and Evaluation Study
Source: JMIR Med Inform. 2026 May 15;14:e80416. doi: 10.2196/80416 (PMC13221620; doi:10.2196/80416)
Supplement: Multimedia Appendix 10 [file medinform_v14i1e80416_app10.docx]

## Multimedia Appendix 10: MA3: Final assessment macro *F_1_*-scores for risk estimation across three risk domains and systems.


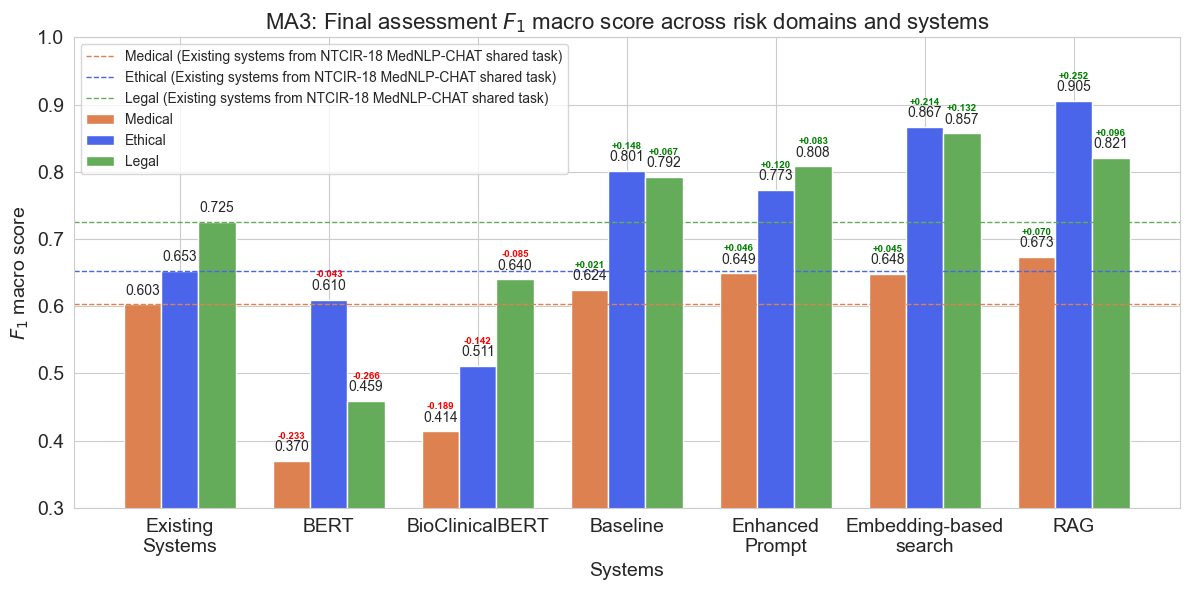


Figure S1. Final assessment (MA3) macro *F*_1_*-*scores for risk classification across three risk domains, four systems: baseline, enhanced prompt, embedding-based search, and RAG, and reruns from strong supervised text classifiers (BERT and BioClinicalBERT). Each bar represents the model’s final assessment performance and improvements over existing systems, as reported in the NTCIR-18 MedNLP-CHAT shared task.
